# A Mutation in the Carbohydrate Recognition Domain Drives a Phenotypic Switch in the Role of Galectin-7 in Prostate Cancer

**DOI:** 10.1371/journal.pone.0131307

**Published:** 2015-07-13

**Authors:** Marilyne Labrie, Maria Vladoiu, Bruno G. Leclerc, Andrée-Anne Grosset, Louis Gaboury, John Stagg, Yves St-Pierre

**Affiliations:** 1 INRS-Institut Armand-Frappier, Laval, Québec, Canada; 2 Centre de Recherche du Centre Hospitalier de l'Université de Montréal (CRCHUM), Montréal, Québec, Canada; 3 Institut de Recherche en Immunologie et Cancérologie, Montréal, Québec, Canada; Innsbruck Medical University, AUSTRIA

## Abstract

The observation that galectin-7 (gal-7) is specifically expressed in mammary myoepithelial (basal) cells prompted us to investigate whether this protein is expressed in the basal cells of other tissues. Given that breast and prostate cancer have remarkable underlying biological similarities and given the important roles of basal cells in prostate cancer, we examined the expression patterns and role of gal-7 in human prostate cancer. Using tissue microarray, we found that although gal-7 is readily expressed in basal cells in normal prostate tissue, it is downregulated in prostate cancer (PCa) cells. *De novo* expression of gal-7 in prostate cancer cells increases their sensitivity to apoptosis in response to etoposide and cisplatin. The assessment of a carbohydrate-recognition domain (CRD)-defective mutant form of gal-7 (R7S) showed that the ability of this protein to modulate apoptosis was independent of its CRD activity. This activity was also independent of its ability to translocate to the mitochondrial and nuclear compartments. However, CRD activity was necessary to inhibit the invasive behaviors of prostate cancer cells. *In vivo*, gal-7 overexpression in PCa cells led to a modest yet significant reduction in tumor size, while its CRD-defective mutant form significantly increased tumor growth compared to controls. Taken together, these results suggest that although *de novo* expression of gal-7 may be an interesting means of increasing the tumorigenic phenotypes of PCa cells, alterations in the CRD activity of this protein drive a phenotypic switch in its role in PCa cells. This CRD-independent activity represents a paradigm shift in our understanding of the functions of galectin. The R74S model will be useful to distinguish CRD-dependent and CRD-independent functions of gal-7 in cancer progression.

## Introduction

Galectin-7 (gal-7) is a p53-induced gene that is mainly expressed in stratified epithelial cells [1, 2]. Its expression can also be induced by other transcription factors, including mutant forms of p53 and CCAAT/enhancer-binding protein beta (C/EBPβ) [3, 4]. Its expression is also regulated by epigenetic mechanisms, including DNA methylation [5, 6]. Its role in UVB-induced keratinocyte apoptosis [7] and in re-epithelialization of corneal wounds [8] support the idea that gal-7 is important for maintaining homeostasis in epithelial cells. Unsuprisingly, a number of studies have shown that dysregulation of gal-7 expression has a strong effect on the progression of multiple types of cancers of epithelial origin. In mammary tissues, for example, gal-7 is specifically expressed in myoepithelial (basal) cells, and its overexpression in breast cancer tissues correlates with resistance to apoptosis and the spread of metastasis to the bone and lung [9]. Overexpression of gal-7 is also associated with poor survival in patients with epithelial ovarian cancer [6, 10] and with malignancy in patients with squamous cell carcinoma of the tongue [11]. These associations between abnormally high levels of gal-7 and poor prognosis are also present in esophageal and hypopharyngeal squamous cell carcinomas [12, 13]. However, as a number of studies have shown, gal-7, similar to other galectins, plays a dual role in cancer and can have a protective role in certain cases, most notably by increasing the sensitivity of cancer cells to pro-apoptotic stimuli and by reducing cell growth and angiogenesis. These activities have been relatively well documented in gastric, urothelial, and colon cancers, as well as in cervical squamous carcinoma [6, 14, 15]. In fact, the observations that genetically engineered cervical, gastric and colon cancer cells overexpressing gal-7 fail to induce gastric tumors in xenografted mice suggest that epigenetic drugs or gal-7-specific gene therapy could be used to suppress the development of specific types of cancer [6, 14, 15]. Given the increasing popularity of epigenetic treatments for cancer, it is thus imperative to determine whether gal-7 has a pro- or anti-tumor function in any given type of cancer, most notably those of epithelial origin.

The various roles of gal-7 in cancers of epithelial origin are currently unclear and may be associated with a variety of factors. One must first consider the importance of the subcellular compartmentalization of gal-7, which has been found in the cytosolic, mitochondrial, and nuclear compartments [15–17]. Gal-3, for example, is able to induce resistance to apoptosis, and this activity depends on its translocation from the cytosol to the mitochondria [18]. Whether such intracellular compartimentalization is also important for gal-7 to regulate apoptosis is unknown. Alternatively, the dual role of gal-7 may depend on its binding partners because it is well known to bind glycosylated proteins via its carbohydrate-recognition domain (CRD). There are increasing indications, however, that galectins also interact with non-glycosylated proteins in a CRD-independent manner [19]. This observation has been well documented for intracellular galectins. The most important feature of intracellular galectins may be their ability to directly bind Bcl-2 family members via a CRD-independent interaction. This activity has been shown for many galectins, including gal-7 [16]. The galectin/Bcl-2 interaction shifts the balance of activity between pro- and anti-apoptotic members of the Bcl-2 family to regulate apoptosis [16, 20, 21]. Other CRD-independent functions of galectins include RNA processing in the nucleus [22] and the regulation of cell cycle progression [23]. All of these CRD-independent functions rely on protein-protein interactions. In fact, certain galectins, such as gal-10, harbor markedly low affinities for galactosides and are believed to act mainly through other factors [24]. These CRD-independent functions represent a paradigm shift in our understanding of galectin function and in the development of galectin-specific inhibitors.

The observation that gal-7 is specifically expressed in epithelial cells, particularly in mammary myoepithelial (basal) cells (but not in luminal cells), prompted us to investigate whether it is expressed in the basal cells of other tissues. Given that breast and prostate cancer have remarkable underlying biological similarities [25] and given the important role of basal cells in prostate cancer [26], we studied the expression pattern and role of gal-7 in human prostate cancer.

## Materials and Methods

### Cell lines and animals

PC3 [27] and DU-145 [28] cell lines were a generous gift from Dr. Benoit Ochietti (McGill University, Montréal, QC), and the LNCaP cells [29] were kindly provided by Dr. Thomas Sandersons (INRS-Institut Armand-Frappier, Laval, QC. The HACAT cell line [30] was provided by Dr. Thierry Magnaldo (Génétique et Physiopathologie des Cancers Épidermiques, Faculté de Médecine, Nice, France). All cell lines used in this study were originally obtained from the American Type Culture Collection (ATCC, Manassas, VA, USA). The DU-145 and HACAT cell lines were maintained in Dulbecco’s modified Eagle’s medium. The PC3 and LNCaP cell lines were maintained in an F-12k nutrient mixture and RPMI 1640 medium, respectively. Culture media were supplemented with 10% (v/v] fetal bovine serum, 2 mmol/L L-glutamine, 10 mmol/L HEPES buffer, and 1 mmol/L sodium pyruvate. All cell culture products were purchased from Life Technologies (Burlington, ON, Canada). NOD/SCID mice were obtained from The Jackson Laboratory. Animals were housed under sterile conditions with *ad libitum* access to food and water. All animal studies were approved by the Institutional Animal Care and Use Committee of the Centre de Recherche du Centre Hospitalier de l’Université de Montréal

### Immunohistochemistry

Tissue microarrays were used to assay 32 tissue samples of prostate adenocarcinoma, 20 hyperplasia, 5 saccular ectasia and 3 cancer-adjacent normal prostate tissues (US Biomax, Rockville, MD, USA and LifeSpan BioSciences, Seattle, WA, USA). Immunostaining reactions for gal-7 were performed using a Discovery XT automated immunostainer (Ventana Medical Systems). Deparaffinized sections were incubated in cell conditioning solution, pH 8.0 (Ventana Medical Systems), for antigen retrieval and then stained for 60 min with an anti-human gal-7 polyclonal antibody (R&D Systems, Minneapolis, MN, USA) diluted 1:150. The slides were counterstained with hematoxylin. The sections were scanned at a high resolution using a Nanozoomer Digital Pathology scanner (Hamamatsu, Bridgewater, NJ).

### Immunofluorescence

Cells were fixed in 3% (w/v) paraformaldehyde for 15 min, permeabilized in 0.1% (v\v) PBS/Triton X-100 for 5 min and blocked overnight at 4°C in 1% (w/v). PBS/BSA (PBA). A goat anti-human gal-7 (diluted 1:750) primary antibody and rabbit anti-goat Alexa Fluor 488 (diluted 1:500) secondary antibody were used (Life Technologies). Filamentous actin was stained with Alexa Fluor 594-conjugated phalloidin (1:500 dilution; Life Technologies). All antisera were diluted in 1% (w/v) PBA, and all washing steps were performed with PBS. Nuclei were stained with ProLong Gold Antifade Reagent with 4',6-diamidino-2-phenylindole (DAPI) (Life Technologies). Cells were visualized under a Carl Zeiss LSM780 confocal microscope, and digitized images were generated using Carl Zeiss ZEN software (Zeiss, Jena, Germany).

### RNA isolation and RT-PCR

Total cellular RNA was isolated from cells using TRIzol reagent (Life Technologies) according to the manufacturer’s instructions. First-strand cDNA was prepared from 2 μg of cellular RNA in a total reaction volume of 20 μL using Omniscript reverse transcriptase (QIAGEN, Mississauga, ON, Canada). After reverse transcription, human *gal-7* (gene ID 3963, sense primer: 5’-TCC CAA TGC CAG CAG GTT CCA TGT-3’ and antisense primer: 5’-GAA GCC GTC GTC TGA CGC GAT GAT-3’) and *GAPDH* (gene ID 2597, sense primer: 5’-CGG AGT CAA CGG ATT TGG TCG TAT-3’ and antisense primer: 5’-CAG AAG TGG TGG TAC CTC TTC CGA-3’) cDNAs were amplified under the following conditions: 94°C for 3 min, followed by 35 cycles at 94°C for 40 sec, 60°C for 40 seconds, and 72°C for 40 seconds and then a final extension step at 72°C for 10 min. PCR was performed in a thermal cycler (MJ Research, Watertown, MA). The amplified products were analyzed on 1% (w/v) agarose gels by electrophoresis followed by gel staining with SYBR Safe (Life Technologies).

### Generation of stable transfectants expressing gal-7

To obtain stable DU-145 transfectants expressing gal-7^wt^ or gal-7^R74S^, cDNAs encoding the wild-type or mutated (R74S) human gal-7 gene were cloned into the Srα eukaryotic expression vector (kindly provided by Dr. François Denis) as previously described [17] Control cells were generated using an empty Srα vector. Transfections were performed with Lipofectamine 2000 according to the manufacturer’s instructions (Life Technologies). After 48 h of culturing, transfected cells were allowed to grow in complete medium containing 2 μg/ml puromycin. Individual colonies were expanded, and gal-7 expression was monitored by western blot analysis. A minimum of two clones of each type was used to confirm the results.

### Western blot analysis

For whole cell extracts, cells were homogenized and resuspended in radio-immunoprecipitation assay (RIPA) buffer (Thermo Fisher Scientific, Ottawa, ON, Canada) containing a cocktail of protease inhibitors (Roche, Mississauga, ON, Canada). Mitochondria and nuclei were isolated using a mitochondrial isolation kit (Thermos Scientific) and nuclear extraction kit (Sigma-Aldrich, Oakville, ON, Canada), respectively, according to the manufacturers’ instructions. Equal amounts of whole-cell, cytoplasmic, mitochondrial or nuclear extracts were separated by SDS-PAGE and transferred onto nitrocellulose membranes (Bio-Rad Laboratories, Mississauga, ON, Canada). The membranes were first blocked with 5% (w/v) milk in PBS/0.5% Tween 20 (v/v) for 60 min at room temperature and subsequently blotted overnight in a solution containing 3% PBA, 0.5% Tween 20 and the following antibodies: a goat anti-mouse gal-7 polyclonal antibody (diluted 1:1000; R&D Systems), a rabbit anti-poly(ADP-ribose) polymerase (Parp)-1 (p25) polyclonal antibody (1:5000; Epitomics, Burlingame, CA, USA), a mouse anti-β-actin (1:20000; Sigma-Aldrich), a rabbit anti-COX IV (1:1000; Cell Signaling Technology, Beverly, MA, USA), a rabbit anti-tubulin (1:1000; Cell Signaling Technology) or a mouse anti-lamin A/C (1:1000; Cell Signaling Technology) antibody. Secondary antibodies included horseradish peroxidase-conjugated donkey anti-rabbit (GE Healthcare, Baie-d'Urfé, QC, Canada), donkey anti-goat (R&D Systems) or sheep anti-mouse (GE Healthcare) IgG. Detection was performed by the enhanced chemiluminescence method (GE Healthcare).

### Electron microscopy

Cells were fixed in a 0.1% (v/v) glutaraldehyde and 4% (w/v) paraformaldehyde solution and embedded in Spurr’s resin. Ultrathin sections were placed on nickel grids and incubated in sodium metaperiodate. Samples were then blocked in 1% PBA for 5 min and incubated for 60 min with a goat anti-human gal-7 polyclonal antibody (1:150) followed by incubation with a rabbit anti-goat 10-nm gold-conjugated secondary antibody (1:20, Electron Microscopy Sciences, Hatfield, PA, USA). The samples were counterstained with uranyl acetate and lead citrate before visualization under a Hitachi H-7100 transmission electron microscope.

### [^3^H]-thymidine incorporation

Proliferation of cells was determined by measuring the incorporation of [^3^H]-thymidine. Cells were seeded in triplicate at a density of 2 x 10^3^ cells/well into a 96-well plate and subsequently incubated with or without 5 μM cisplatin for 96 h. After 80 h of incubation, 1 μCi of [^3^H]-thymidine was added to each well. At the end of the incubation period, the cells were harvested with a semiautomatic cell harvester (Skatron Instruments, Lier, Norway) and transferred onto a Printed Filtermat A (Wallak, Turky, Finland). Incorporated radioactivity was determined using a RackBeta (LKB, Turky, Finland) scintillation counter.

### Invasion assay

Serum-induced cell invasion was examined using a 24-well Matrigel invasion chamber (BD Biosciences, Mississauga, ON, Canada) with an 8 μm-pore membrane. A total of 5 x 10^4^ cells were incubated within the upper chamber in serum-free medium. The lower chamber contained medium supplemented with 10% fetal bovine serum. After 24 h of incubation, the upper surface of the insert was wiped gently with a cotton swab to remove the non-migrating cells. Cells that had migrated to the lower surface of the membrane were stained with toluidine blue and counted separately by microscopy.

### Scratch wound healing assay

Confluent monolayers were obtained by seeding 1 x 10^5^ cells onto a 24-well plate the day before the experiment. A scratch was made with a pipet tip in the cell monolayer, followed by washing with PBS to remove cell debris. Immediately after and 24 h after the PBS wash step, the microscopic fields were photographed, and the scratch width was measured using Image J software. For live cell imaging, one day prior to the experiment, 4 x 10^5^ cells were seeded onto a 6-well glass-bottom culture plate (MatTek Corporation, Ashland, MA, USA). After the scratch was made, the plate was moved to a PM S1 incubator, and the migration was visualized under a Carl Zeiss LSM780 confocal microscope (Carl Zeiss, Toronto, ON, Canada). Images were captured every 10 min for 2 h. For each cell type, the movements of 30 separate cells were measured. Cell movement was analyzed using the following Image J plugins: manual tracking and chemotaxis tool.

### Cell proliferation assay

As a control for cell proliferation during the invasion assay and scratch wound healing test, a total of 2.5 x 10^4^ cells were seeded onto a 12-well plate (Fisher Scientific). At the indicated times, the cells were washed with PBS, trypsinized, stained with trypan blue and counted using a hemocytometer.

### Production of recombinant proteins

Gal-7 cDNA was cloned into pET-22b(+) using the NdeI and HindIII restriction enzymes. The protein was produced in *E*. *coli* BL21 (DE3) at 37°C. Isopropyl β-D-1-thiogalactopyranoside (*IPTG*) (1 mM) was added to the bacterial culture at an OD_600nm_ of 0.6–0.7, and the bacteria were further incubated for 4 h. Bacterial pellets were resuspended in lysis buffer (0.7 mg/mL lysozyme, 10 mM Tris, pH 8, 100 mM NaCl, 1 mM EDTA, 1 mM DTT and protease inhibitor cocktail), incubated for 1 h at 37°C and centrifuged for 30 min at 15,000 rpm (4°C). The supernatant was then filtered and applied to a lactose-agarose column, and the protein was eluted in 1-mL fractions with a 150-mM lactose solution. Purified fractions were analyzed by SDS-PAGE. Gal-7 was dialyzed against 20 mM potassium phosphate at pH 7.2 for all subsequent experiments.

### Glycan array

A mammalian glycan array (V5.2) was performed by the Consortium for Functional Glycomics (CFG). Briefly, recombinant gal-7^wt^ and gal-7^R74S^ proteins were conjugated to FITC and tested against version 5.2 of the printed array. This array consisted of 609 glycans in replicates of 6. The lists of the glycans and their linkers used in the different versions of the array can be found at http://www.functionalglycomics.org/static/consortium/resources/resourcecoreh.shtml. FITC-conjugated gal-7 was incubated with the sugars, and relative fluorescence units (RFUs) were measured. To eliminate some of the false hits that contained a single very high or low point, the highest and lowest points from each set of six replicates were removed. Consequently, the averages include 4 values rather than 6.

### Binding assay

Briefly, a fluorescein isothiocyanate (FITC)/DMSO solution was added to recombinant gal-7 in a 0.1-M NaHCO_3_ (pH 9.2) solution and then incubated for 2 h at room temperature on a roller. FITC-conjugated gal-7 was then purified using a PD-10 sepharose column (GE Healthcare) and eluted with PBS containing 0.01% (v/v] sodium azide. To measure FITC-gal-7 binding to the cell surface, 2.5 x 10^5^ cells were incubated for 30 min with the indicated concentrations of gal-7 and then washed twice with PBS and resuspended in 500 μl PBS. For competition assays, 0.1 M β-lactose was added to cells, which were then incubated with FITC-conjugated gal-7. Samples were analyzed by FACSCalibur (BD Biosciences) and Flowing Software.

### *In vivo* experiments

A mix of 3 independent clones (2 x 10^6^ cells) for each transfectant was injected subcutaneously into 6-week-old NOD/SCID mice. Tumor measurements were obtained twice a week. On day 61, the mice were sacrificed, and the primary tumors were harvested and snap-frozen in liquid nitrogen.

### Statistical analysis

Statistical significance of the experiments was evaluated using unpaired Student's t-tests. The results were considered statistically significant at P ≤ 0.05.

## Results

### Gal-7 expression in human prostate tissues and cancer cell lines

**Fig 1:**
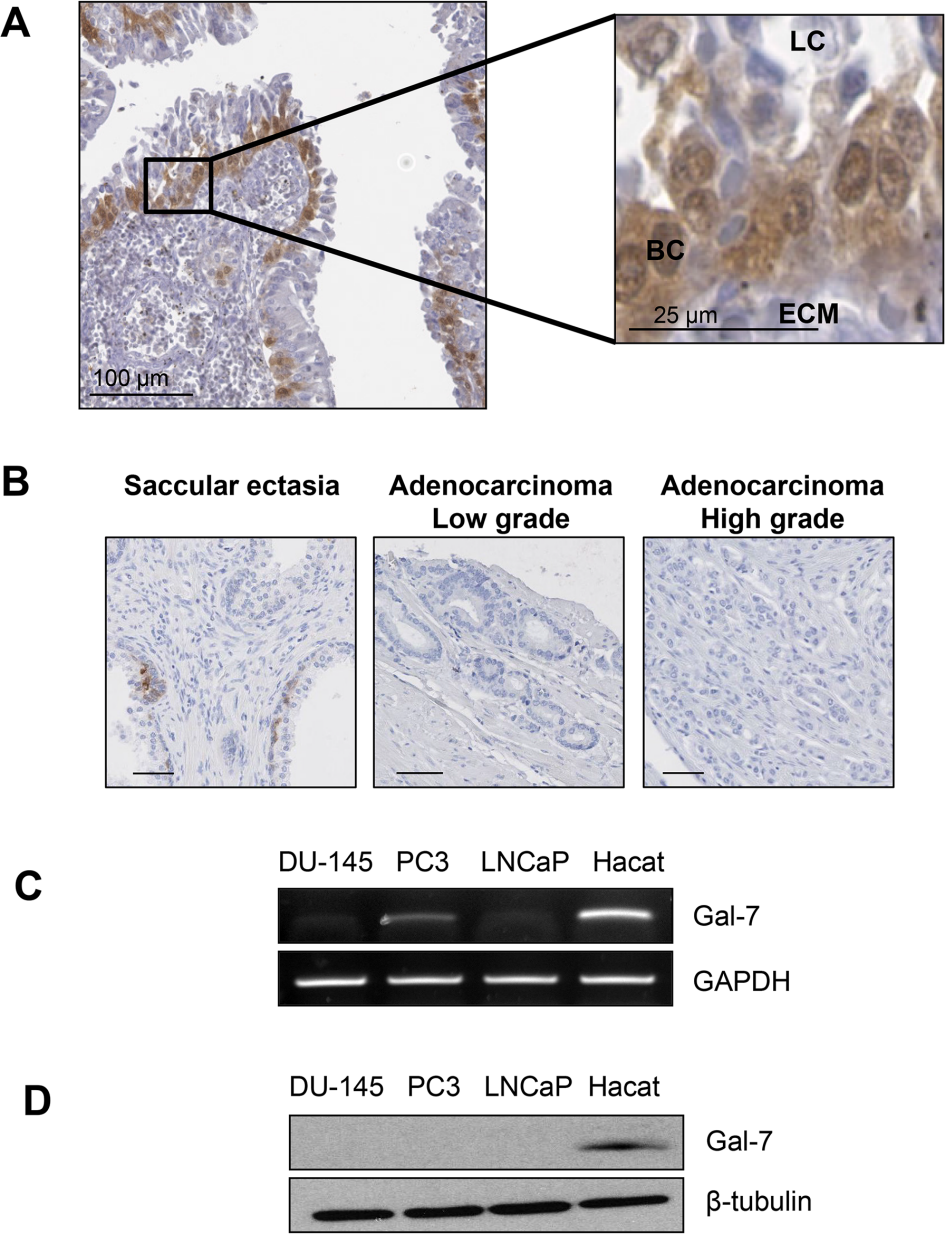
Gal-7 expression in prostate tissues and cancer cell lines. Immunohistochemical (IHC) staining of gal-7 in 3-μm thick sections of formalin-fixed paraffin-embedded (**A**) healthy prostate tissues and (**B**) tissues associated with pathological disorders of the prostate. (**C**) Semi-quantitative RT-PCR and (**D**) western blot analysis of gal-7 expression in DU-145, PC3 and LNCaP human prostate cancer cell lines. The HaCaT keratinocyte cell line was used as a positive control. GAPDH and β-tubulin were used as loading controls. LC: luminal cells, BC: basal cells, and ECM: extracellular matrix.

Gal-7 expression in human prostate tissues has not previously been reported. Using immunohistochemistry (IHC), we first investigated the expression pattern of gal-7 in normal prostate tissues. Our results revealed strong nuclear and cytoplasmic expression in the basal cell layers of normal prostate glands, with no staining observed in the luminal epithelial cells (Fig 1A). We then investigated the presence of gal-7 in various types of prostate malignancies (including 32 prostate adenocarcinomas) using commercial tissue microarrays and found negligible gal-7 protein expression in the tumor tissues (Fig 1B and 1C). The low expression in PCa tissues was consistent with expression patterns found during our investigations of gal-7 expression at the mRNA and protein levels in the most common prostate cancer cell line models. Immunoblotting experiments demonstrated that none of these cell lines expressed readily detectable levels of gal-7; however, mRNA was expressed at a low but detectable level in PC3 cells (Fig 1). Taken together, these results showed that although gal-7 is readily expressed in basal cells within normal prostate tissue, it is completely absent in prostate cancer cells.

### Production and characterization of wild-type and CRD-defective gal-7 protein

**Fig 2:**
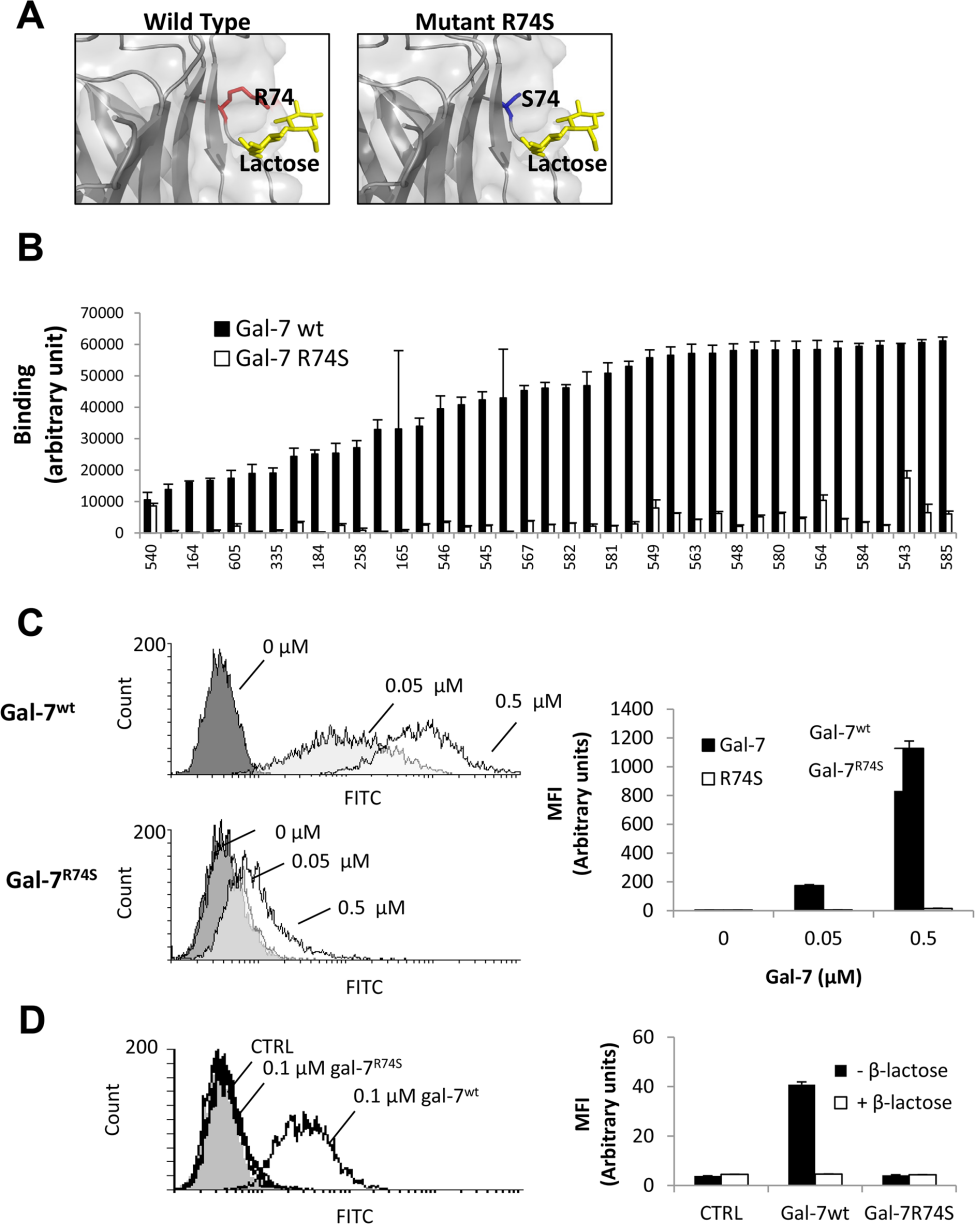
WT and R74S-mutated gal-7 CRD activity. (**A**) 3D view of the gal-7^wt^ and gal-7^R74S^ CRDs in the presence of lactose. (**B**) A glycan array was used to verify the binding of gal-7^wt^ and gal-7^R74S^ to a large variety of sugars. The graph depicts the binding of gal-7^wt^ and gal-7^R74S^ to the sugars. Only RFUs larger than 10,000 are presented. The sugar names are listed in S1 Table. The error bars represent SDs (n = 6). Flow cytometry analysis showing binding of gal-7^wt^ and gal-7^R74S^ to the surfaces of DU-145 cells (**C**) in the absence or (**D**) presence of 0.1 M β-lactose. Binding assays were conducted using the indicated concentrations of FITC-labeled recombinant gal-7. MFI: mean fluorescence intensity. The results represent three independent experiments.

To investigate the functions of gal-7 in prostate cancer cells and to determine the importance of its CRD, we generated a series of stable transfectants expressing wild-type gal-7 and a mutated form (R74S) of the protein (Fig 2A). This mutation is known to inhibit the ability of gal-7 to bind lactose and to reduce its translocation from the cytosol to the mitochondria and/or the nucleus [17]. Our previous analysis using solution NMR spectroscopy showed that the R74S mutation induced only limited and local changes in gal-7 folding. To further determine the extent to which the CRD of gal-7 is disrupted by the R74S mutation, we compared its binding properties to those of wild-type gal-7 using a CFG glycan array (version 5.2) [31]. The entire list of glycans tested and the results of the binding assays can be found online (http://functionalglycomics.org/). Our results confirmed that the R74S mutation suppressed CRD activity independent of the glycan motifs assessed (Fig 2B, S1 Table). This suppression of CRD activity was confirmed by flow cytometric analysis of the binding of FITC-labeled recombinant gal-7^wt^ and gal-7^R74S^ at the surfaces of DU-145 prostate cancer cells (Fig 2C and 2D). Taken together, these results demonstrate that R74S abolishes the CRD activity of human gal-7.

### Characterization of intracellular localization of wild-type gal-7 and gal-7^R74S^

**Fig 3:**
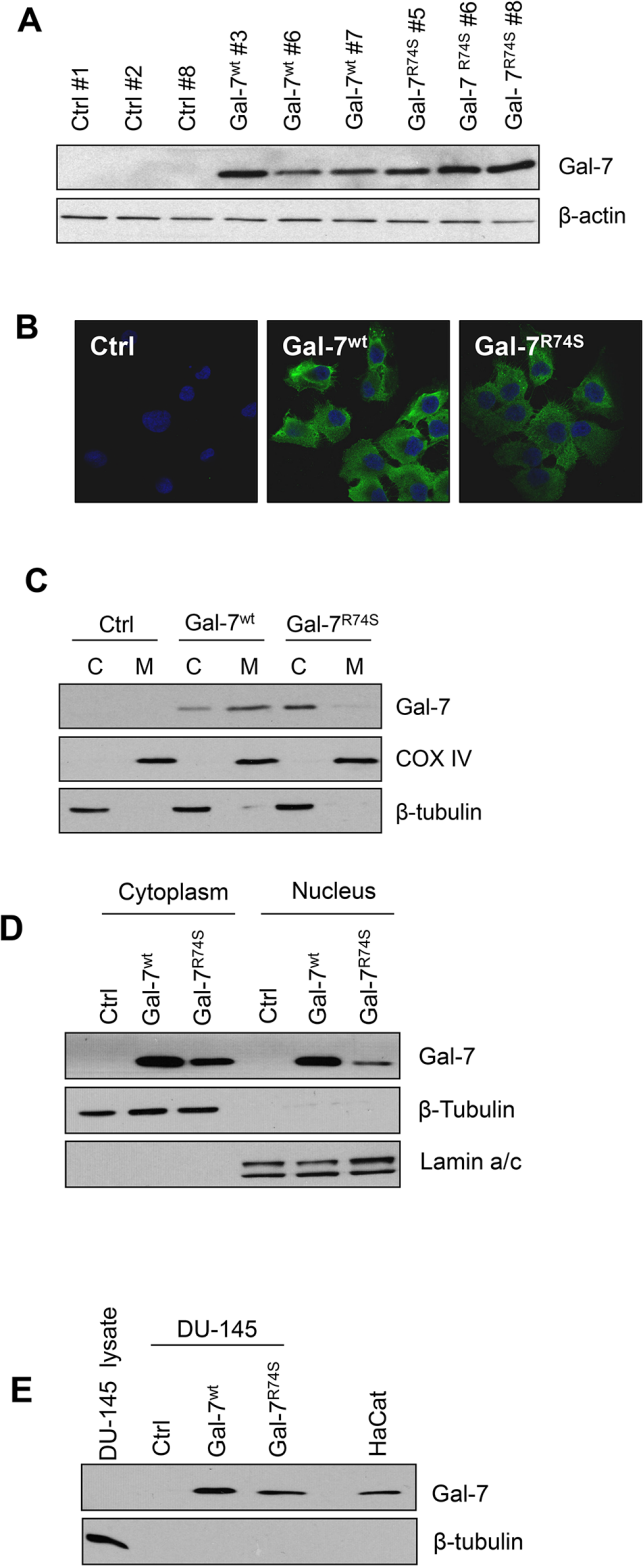
Intracellular localization of wild-type and R74S-mutated gal-7 proteins in DU-145 cells. (**A**) Western blot analysis showing the expression of gal-7 in various stable DU-145 clones transfected with expression vectors encoding wild-type and mutated gal-7. Controls included cells transfected with empty Srα vectors. β-actin was used as a loading control. (**B**) Confocal imaging showing the intracellular distribution of gal-7 in DU-145 transfectants. (**C-D**) Western blot analysis showing gal-7 expression in cytosolic, nuclear and mitochondrial fractions prepared from DU-145 cells. β-tubulin, COX IV and lamin A/C were used as cytosolic, mitochondrial and nuclear markers, respectively. (**E**) Western blot analysis showing the secretion of gal-7 in the extracellular media of DU-145 cells expressing gal-7^wt^ or gal-7^R74S^. β-tubulin expression was monitored to exclude the possibility of cell lysis. Intracellular protein extracts from control DU-145 cell and HaCaT cell supernatants were used as positive controls for β-tubulin expression and gal-7 secretion, respectively. All results represent three independent experiments, including a minimum of two independent DU-145 clones.

To investigate the role of gal-7 and its CRD in prostate cancer, DU-145 transfectants expressing either gal-7^wt^ or gal-7^R74S^ were generated. Control transfectants (generated using empty expression vectors) did not express detectable levels of gal-7 (Fig 3A and 3B). Immunoblotting of mitochondrial and nuclear enriched fractions showed detectable expression of gal-7^wt^ in the cytosol, mitochondria and nuclei of DU-145 cells (Fig 3C and 3D). In contrast, there was no detectable expression of gal-7 in the mitochondria of transfectants expressing gal-7^R74S^. Both proteins were found in the extracellular media of the cells (Fig 3E). Electron microscopy analysis of the DU-145 cells confirmed the expression of gal-7^wt^ in the cytosol, nucleus, and mitochondrial outer membrane, while the expression of gal-7^R74S^ was restricted to the cytosol (S1 Fig). Interestingly, electron microscopic analysis revealed the presence of gal-7^wt^- and gal-7^R74S^-rich protrusions at the cytoplasmic membrane (S1 Fig) consistent with previous reports, suggesting that gal-7 associates with the actin cytoskeleton to regulate cell motility [10, 32, 33].

### Gal-7 promotes apoptosis in prostate cancer cells

**Fig 4:**
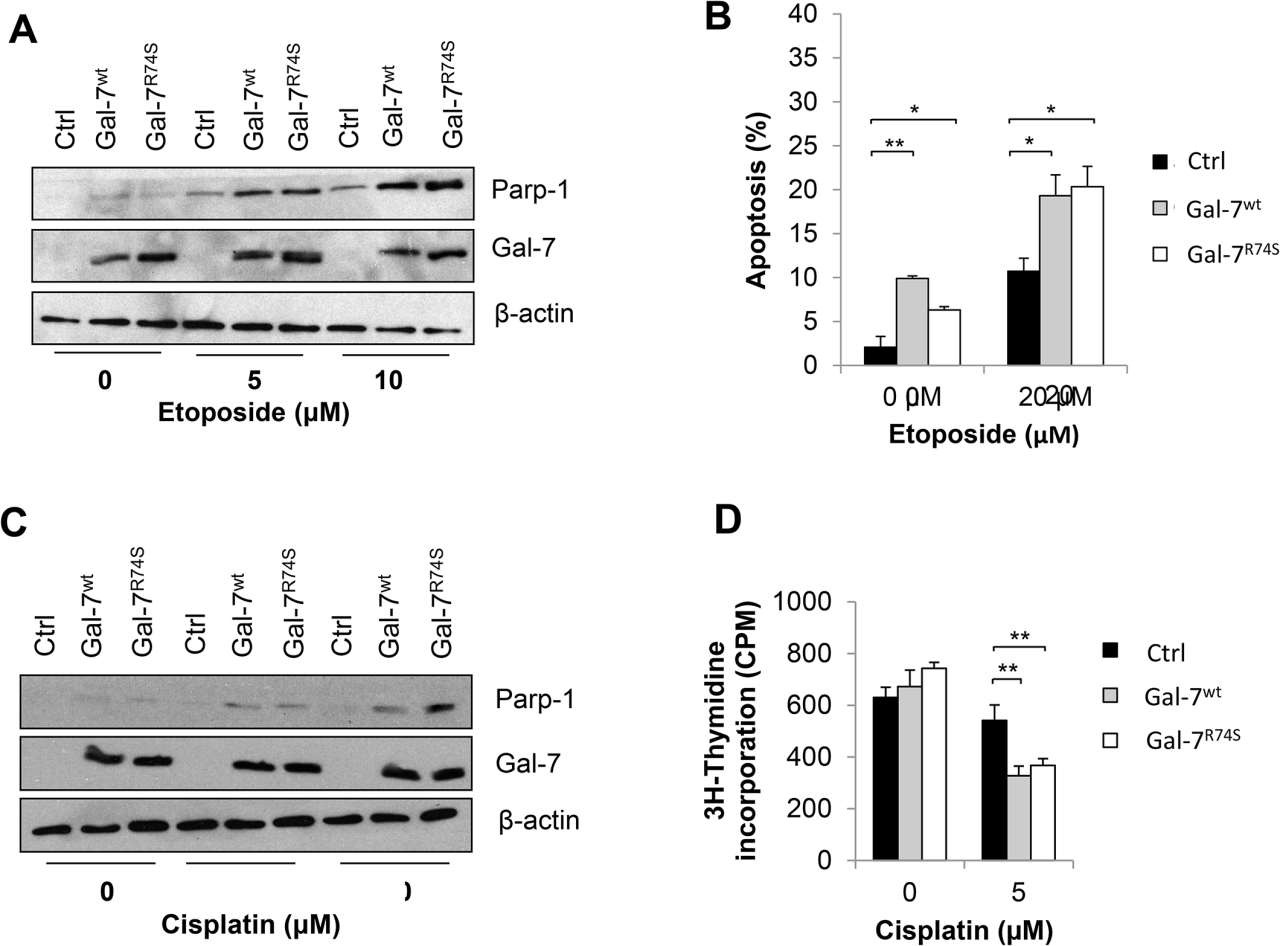
Gal-7 increases the sensitivity of DU-145 cells to apoptosis. (**A**) Cells were incubated for 16 h with the indicated concentrations of etoposide and tested for apoptosis by measuring Parp-1 cleavage by western blot. (**B**) Apoptosis was confirmed by counting the number of cells with a fragmented nucleus visualized by DAPI staining. (**C**) Analysis of Parp-1 cleavage in DU-145 cells treated for 16 h with the indicated concentration of cisplatin. (**D**) Cell proliferation of DU-145 cells treated with or without 5 μM cisplatin measured by (^3^H]-thymidine incorporation. All results represent three independent experiments, including a minimum of two independent DU-145 clones. *P ≤ 0.05, **P ≤ 0.01, and ***P ≤ 0.001.

Several studies have reported that ectopic expression of gal-7 renders cervical, gastric and colon cancer cells more sensitive to apoptosis induced by pro-apoptotic drugs [15]. To determine whether this finding is also true in prostate cancer cells, DU-145 transfectants were treated with increasing doses of pro-apoptotic drugs and analyzed for cleavage of Parp-1, which is a commonly used marker of apoptosis [34]. Our results showed that ectopic expression of gal-7 increased the cleavage of Parp-1 induced by etoposide compared to the control cells (Fig 4A). Similar results were obtained when the fragmentation of nuclei in apoptotic cells was visualized with DAPI staining (Fig 4B). The ability of gal-7 to increase the sensitivity of DU-145 cells to apoptosis was also observed using cisplatin as a pro-apoptotic drug (Fig 4C). Interestingly, gal-7^R74S^ was as effective as gal-7^wt^ in increasing the sensitivity of DU-145 to both pro-apoptotic drugs. In both cases, the intracellular localization of gal-7 remained unchanged during the induction of apoptosis (S2 Fig). Using a (^3^H]-thymidine incorporation assay, we also measured proliferation of the transfectants in the absence or presence of cisplatin. We found that both gal-7^wt^- and gal-7^R74S^-expressing DU-145 cells proliferated at the same rates compared to control cells under normal conditions but proliferated more slowly in presence of cisplatin (Fig 4D), which is consistent with the ability of gal-7 to promote drug-induced apoptosis. Taken together, these results show that gal-7 sensitizes DU-145 cells to pro-apoptotic agents independent of its CRD activity and its intracellular compartmentalization.

### Gal-7 but not R74S reduces invasive behaviors of DU-145 cells

**Fig 5:**
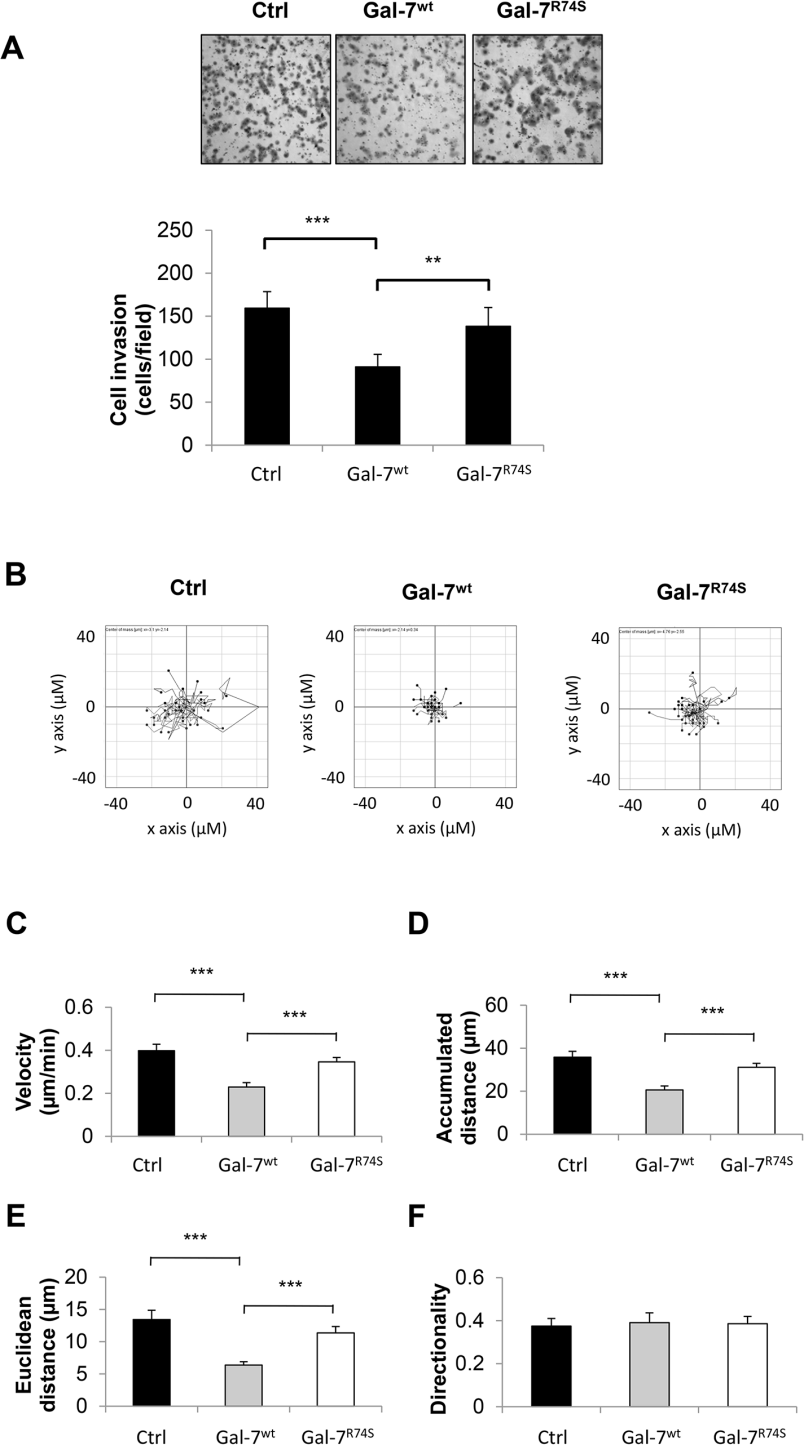
Invasive behavior of DU-145 expressing gal-7^wt^ or gal-7^R74S^. (**A**) Matrigel invasion assay of DU-145 stable transfectants. Cells were seeded into serum-free medium in the upper chamber, and serum was added to the lower chamber. After 16 h, cells that migrated through the Matrigel and the 8 μm-pore membrane were stained and counted. The results represent the number of cells per microscopic field. (**B**) Plots of 30 cells/sample tracked by live cell imaging during the scratch wound healing test. DU-145 cells were seeded onto a 6-well glass-bottom culture plate. A scratch was made, and images were captured every 10 min for 2 h. Quantifications of the (**C**) velocity, (**D**) accumulated distance, (**E**) Euclidean distance and (**F**) directionality are shown. Error bars represent the SEM. All results represent three independent experiments, including a minimum of two independent DU-145 clones. **P ≤ 0.01 and ***P ≤ 0.001.

We next investigated whether gal-7 can modulate the invasive behaviors of DU-145 cells using a standard *in vitro* Matrigel invasion assay. We found that ectopic expression of gal-7^wt^ significantly reduced the invasive behaviors of DU-145 cells compared to control cells lacking gal-7 (Fig 5A). A similar difference was not observed for DU-145 cells expressing gal-7^R74S^. Because cell invasive behaviors might be affected by cell motility, we used live cell imaging to measure cell movements during a scratch wound healing assay (Fig 5B). Our results showed that DU-145 cells expressing gal-7^wt^ significantly reduced cell velocity compared to control cells lacking gal-7 or cells expressing gal-7^R74S^ (Fig 5C). These gal-7^wt^-expressing cells also had lower accumulated and Euclidean distances of migration (Fig 5D and 5E). The directionality of the DU-145 cells was not affected by expression of the gal-7^wt^ or gal-7^R74S^ proteins (Fig 5F). The use of a standard scratch wound healing assay further confirmed that gal-7^wt^ reduced the cell motility of the DU-145 cells. Again, a similar effect was not observed for the gal-7^R74S^ mutant (S3 Fig). No differences in cell proliferation were observed between the cells expressing gal-7^wt^ or gal-7^R74S^ and the control cells (S4 Fig). The addition of recombinant gal-7^wt^ and gal-7^R74S^ to DU-145 cells had no effect, suggesting that extracellular gal-7 is not involved in reducing invasive behaviors (S5 Fig). Taken together, these results indicate that intracellular gal-7 reduces the invasive behaviors of prostate cancer cells by impairing cell motility in a CRD-dependent manner.

### Gal-7 CRD disruption increases tumor growth *in vivo*

**Fig 6:**
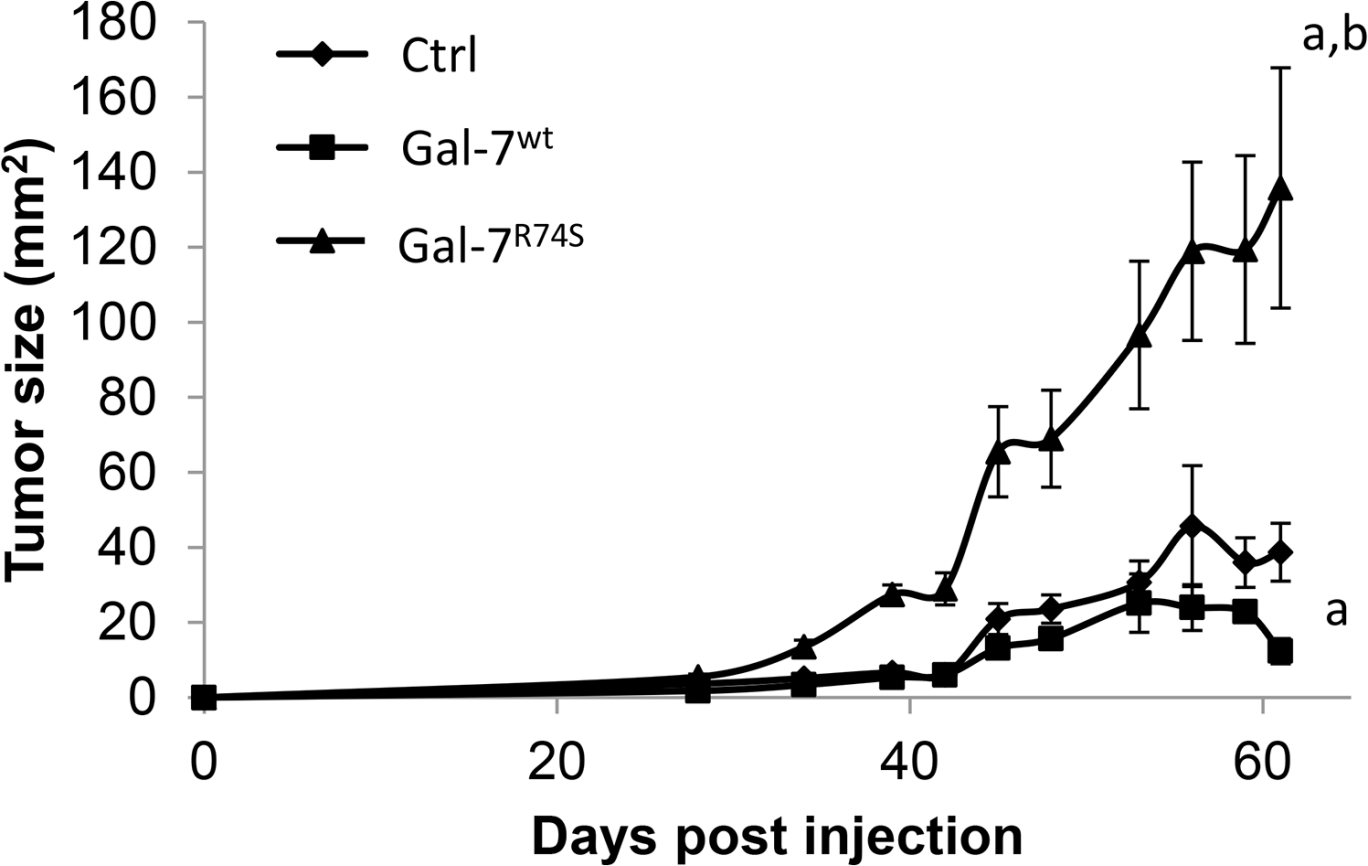
Gal-7^wt^ and gal-7^R74S^ effects on tumor growth. NOD/SCID mice (n = 6) were injected subcutaneously with control DU-145 transfectants or with DU-145 transfectants expressing gal-7^wt^ or gal-7^R74s^. Tumor growth was monitored twice a week for 61 days. N = 6 mice per group.

We next investigated whether gal-7^wt^ and gal-7^R74S^ impact tumor growth using adult male NOD/SCID mice. Mice were injected subcutaneously with DU-145 transfectants, and tumor size was measured twice a week for 61 days, at which time the tumors were harvested to confirm the expression of gal-7 in the gal-7^wt^- and gal-7^R74S^-expressing cells (S6 Fig). Our results showed that the overexpression of gal-7^wt^ led to a modest yet significant (*p* ≤ 0.05) reduction in tumor size (Fig 6). Interestingly, the expression of gal-7^R74S^ caused a significant (*p* ≤ 0.001) increase in tumor growth compared to both the control and gal-7^wt^-expressing cells.

## Discussion

Our previous results showing that gal-7 is specifically expressed in mammary myoepithelial (basal) cells but not in mammary luminal cells prompted us to investigate whether this molecule is expressed in the basal cells of prostate tissues. Using an anti-gal-7-specific Ab, we found that gal-7 immunostaining in human prostate tissues was consistently strong in the nuclei and cytoplasm of prostate basal cells, with the luminal cells showing no detectable staining. This pattern of expression is thus clearly distinct from those reported for gal-1 and gal-3. Indeed, gal-3 is expressed in luminal cells but not in basal cells, while gal-1 is expressed in the endothelial and stromal fibromuscular cells of the prostate [35]. This distinct expression pattern for gal-7 is also observed in prostate cancer cell lines. Although we found no detectable expression of gal-7 in the prostate cancer cell lines tested, gal-3 has been shown to be readily expressed in both PC-3 and DU-145 cells [36]. Gal-3 expression is also reduced in PCa cells compared with normal prostate cells but is still detectable by IHC in a significant number of samples [36, 37]. Our data, however, clearly showed that gal-3 and gal-7 had distinct properties in PCa cells. For example, in contrast with gal-7, cytoplasmic gal-3 increased Matrigel invasion and cell growth while decreasing apoptosis induction, and nuclear expression had a completely opposite effect [38]. Thus, gal-3 and gal-7 possess completely opposite biological activities in PCa cells. Although future experiments will be needed to confirm these results in other prostatic cell lines (including benign cell lines) and other preclinical PCa models. It is important to note that we cannot not use the PC3 cells (another classical cell model of human prostate cancer) because these cells express low but significant endogenous galectin-7 (as shown in Fig 1). We cannot use the LNCaP cell model (another commonly used model) because this model in androgen-dependent, in contrast to DU-145. Nevertheless, our findings may have important implications in the development of CRD-specific inhibitors against gal-3 and emphasize the need to develop inhibitors that are highly specific for a given galectin.

Our results showed that gal-7 reduced the invasive behaviors of prostate cancer cells by inhibiting their motility. This phenotype is consistent with the localization of this molecule in lamellipodia and filopodia. In normal cells, gal-7 is also found in motility structures, such as podosomes and primary cilia [32, 33]. Interestingly, a mutation at position 74 completely abolished this activity. In contrast, this mutation did not affect the ability of gal-7 to induce apoptosis, indicating that both functions are mediated by distinct gal-7 sites. One possibility is that gal-7 regulates the stability and/or localization of proteins, such as β-catenin, and that the mutation at position 74 abolishes this interaction. In the cytoplasm, β-catenin is either ubiquitinated for proteasomal degradation or localized at cell-cell contact sites, stabilizing E-cadherin and affecting motility [39]. This interaction between galectins and β-catenin has been reported previously [40–42].

We found that gal-7 overexpression sensitized DU-145 prostate cancer cells to apoptosis induced by cisplatin or etoposide. The dual role of gal-7 in apoptosis has been well documented. Because gal-7 binds to bcl-2, our initial hypothesis was that mitochondrial gal-7 could be responsible for this dual role. However, this assumption is clearly not true because cytoplasmic gal-7^R74S^ displays similar pro-apoptotic functions as the wild-type protein. This similarity has also been reported for the anti-apoptotic functions of gal-7 in breast cancer cells [17]. It is indeed quite clear that although gal-7 is expressed in basal cells in normal prostatic and mammary tissues, it plays a completely different role in prostate and breast cancer. The mechanisms leading to the anti- and pro-tumorigenic functions of cytoplasmic gal-7 remain unknown. One possible mechanism involves modulation of the JNK1 pathway, as suggested by Kuwabara *et al*. [14], who showed that the induction of apoptosis by gal-7 in HeLa cells is correlated with activation of this signaling pathway. It is also possible that gal-7 regulates apoptosis by interacting with bcl-2. Other studies have indeed shown that gal-7 interacts directly with Bcl-2 [16]. Cytoplasmic gal-7 could sequester bcl-2 in the cytoplasm, thereby inhibiting its anti-apoptotic function. Alternatively, given the structural homology among members of the bcl-2 family [43], it is possible that gal-7 binds to other bcl-2 structural homologs, thereby altering the delicate balance between pro- and anti-apoptotic proteins. We are currently investigating these possibilities.

The dual role of galectins in modulating tumor progression has been previously noted but is still unclear. Our data showing that gal-7^R74S^ acts as a tumor suppressor *in vitro* and as a pro-tumorigenic protein *in vivo* suggest that the roles of galectins in cancer likely involve a delicate balance between pro- and anti-tumoral interactions occurring within and outside cancer cells, i.e., in the tumor microenvironment. Because gal-7^R74S^ sensitized DU-145 cells to apoptosis without affecting their invasive behaviors or proliferation, leading to the augmentation of tumor growth *in vivo*, it is clear that alterations in the CRD of gal-7 that shift the balance towards CRD-independent binding partners not only have a profound effect on its intracellular distribution in cancer cells but also drive a phenotypic switch in its role in cancer. *In silico* analysis using a publically available dataset, the cBioPortal for Cancer Genomics, shows that the gene encoding human gal-7 is rarely (less than 1% in prostate adenocarcinoma) mutated in PCa and is not mutated within the CRD-coding region. Similar results were obtained with the COSMIC database (one missense mutation out of 528 cases). This indicates that loss of gal-7 expression in PCa cells is probably due to an epigenetic mechanism and/or to the depletion of basal cells. Our data add, however, a new dimension to the role of galectin CRDs in cancer, emphasizing the use of highly specific inhibitors to target members of the galectin family. The identification of important CRD-independent functions represents a paradigm shift in our understanding of galectin functions. Future investigations will be needed to identify in detail the CRD-independent binding partners involved. The availability of our gal-7^R74S^ model will be useful in this regard.

## Supporting Information

S1 Fig Electron microscopic analysis of gal-7^wt^ and gal-7^R74S^ intracellular localization. Electron microscopic analysis of gal-7 and R74S protein distributions in the cytosol, nuclei, mitochondria and protrusions localized to the cytoplasmic membranes of DU-145 transfectants. Bars represent 100 nm.
(TIFF)

S2 Fig Intracellular localization of gal-7 during apoptosis. Western blot analysis showing gal-7 expression in cytosolic, (**B**) mitochondrial and (**C**) nuclear fractions prepared from DU-145 cells treated for 16 h with 10 μM etoposide. β-tubulin, COX IV and laminin C were used as cytosolic, mitochondrial and nuclear markers, respectively. All results represent three independent experiments, including a minimum of two different DU-145 clones.
(TIFF)

S3 Fig Scratch wound healing assay of DU-145 transfectants. DU-145 transfectants were seeded into a 24-well plate. A scratch was made with a pipet tip, photos were taken at time 0 and at 24 h, and the width of the scratch was measured. The results represent the distances of the migration of the cells, and the bars represent the SDs. The results represent three independent experiments. ***P ≤ 0.001.
(TIFF)

S4 Fig Gal-7^wt^ and gal-7^R74S^ effects on cell proliferation. As a control for cell proliferation, cells were seeded into a 24-well plate and counted at the indicated time using trypan blue staining. The results represent three independent experiments.
(TIFF)

S5 Fig Extracellular gal-7 effect on cell motility. (**A**) Plots of 30 cells/sample tracked by live cell imaging during a scratch wound healing assay. DU-145 cells were seeded onto a 6-well glass-bottom culture plate and treated with the indicated concentration of gal-7^wt^. A scratch was made, and images were captured every 10 min for 2 h. Quantifications of the (**B**) velocity, (**C**) accumulated distance, (**D**) Euclidean distance and (**E**) directionality are shown. The error bars represent the SEMs. All results represent three independent experiments, including a minimum of two independent DU-145 clones.
(TIFF)

S6 Fig Gal-7 mRNA expression in DU-145 tumors. NOD/SCID mice (n = 6) were injected subcutaneously with DU-145 transfectants expressing the empty vector or gal-7^wt^/gal-7^R74s^. On day 61, the mice were sacrificed, and the primary tumors were harvested and snap-frozen in liquid nitrogen. Tumor mRNA was extracted and gal-7 expression was measured by RT-PCR. N = 6 mice per group.
(TIFF)

S1 Table Glycan array. The names of the different sugars used in the glycan array are listed. Only those sugars for which gal-7^wt^ had an RFU of larger than 10,000 are presented.
(TIFF)
