# Supplementary figures and images for: A Mutation in the Carbohydrate Recognition Domain Drives a Phenotypic Switch in the Role of Galectin-7 in Prostate Cancer
Source: PLoS One. 2015 Jul 13;10(7):e0131307. doi: 10.1371/journal.pone.0131307 (PMC4500561; doi:10.1371/journal.pone.0131307)

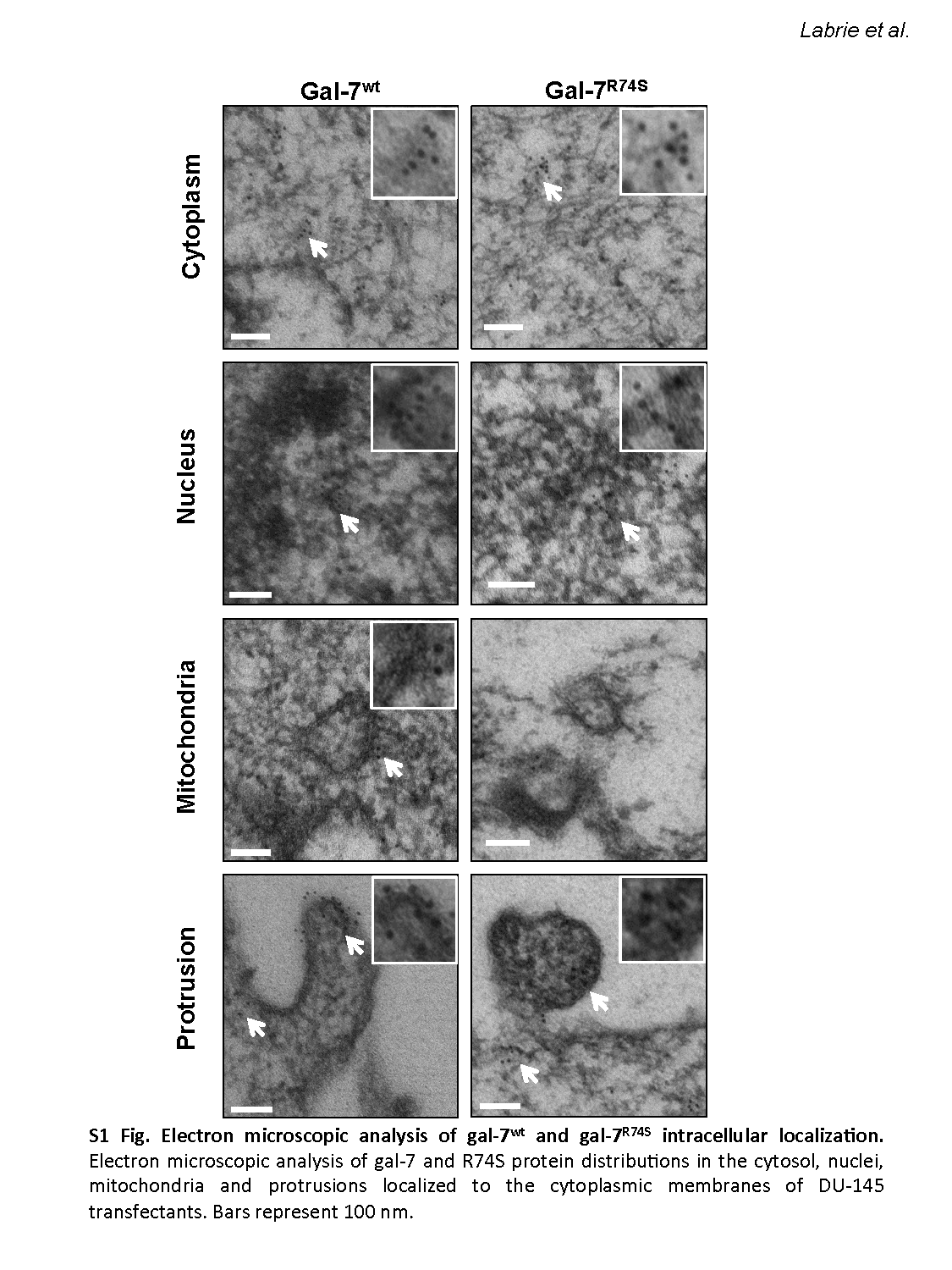

Supplement: S1 Fig — Electron microscopic analysis of gal-7 and R74S protein distributions in the cytosol, nuclei, mitochondria and protrusions localized to the cytoplasmic membranes of DU-145 transfectants. Bars represent 100 nm. (TIFF) [file pone.0131307.s001.tiff]

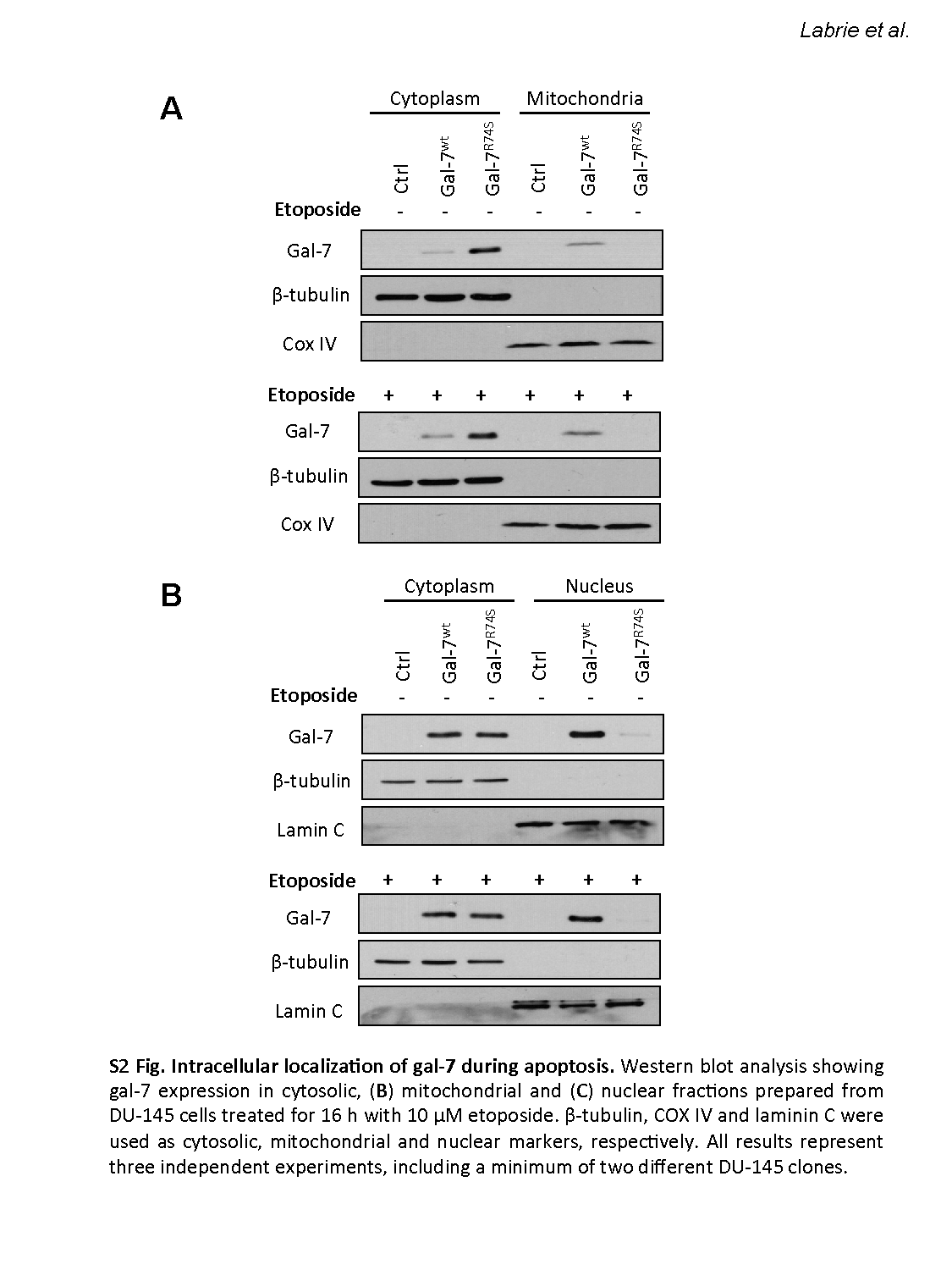

Supplement: S2 Fig — Western blot analysis showing gal-7 expression in cytosolic, (B) mitochondrial and (C) nuclear fractions prepared from DU-145 cells treated for 16 h with 10 μM etoposide. β-tubulin, COX IV and laminin C were used as cytosolic, mitochondrial and nuclear markers, respectively. All results represent three independent experiments, including a minimum of two different DU-145 clones. (TIFF) [file pone.0131307.s002.tiff]

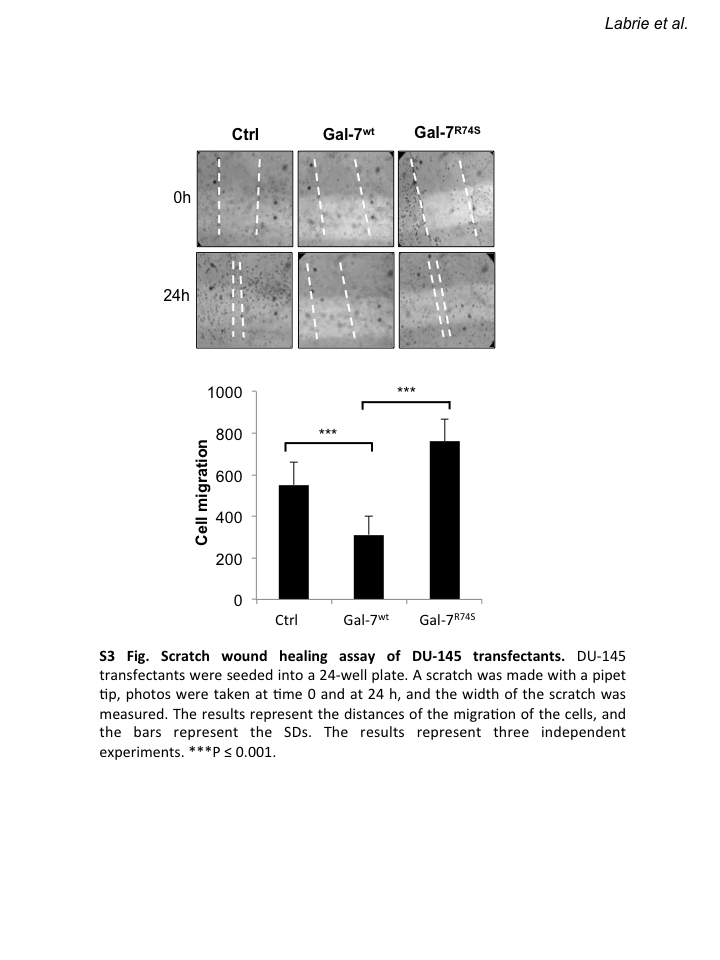

Supplement: S3 Fig — DU-145 transfectants were seeded into a 24-well plate. A scratch was made with a pipet tip, photos were taken at time 0 and at 24 h, and the width of the scratch was measured. The results represent the distances of the migration of the cells, and the bars represent the SDs. The results represent three independent experiments. ***P ≤ 0.001. (TIFF) [file pone.0131307.s003.tiff]

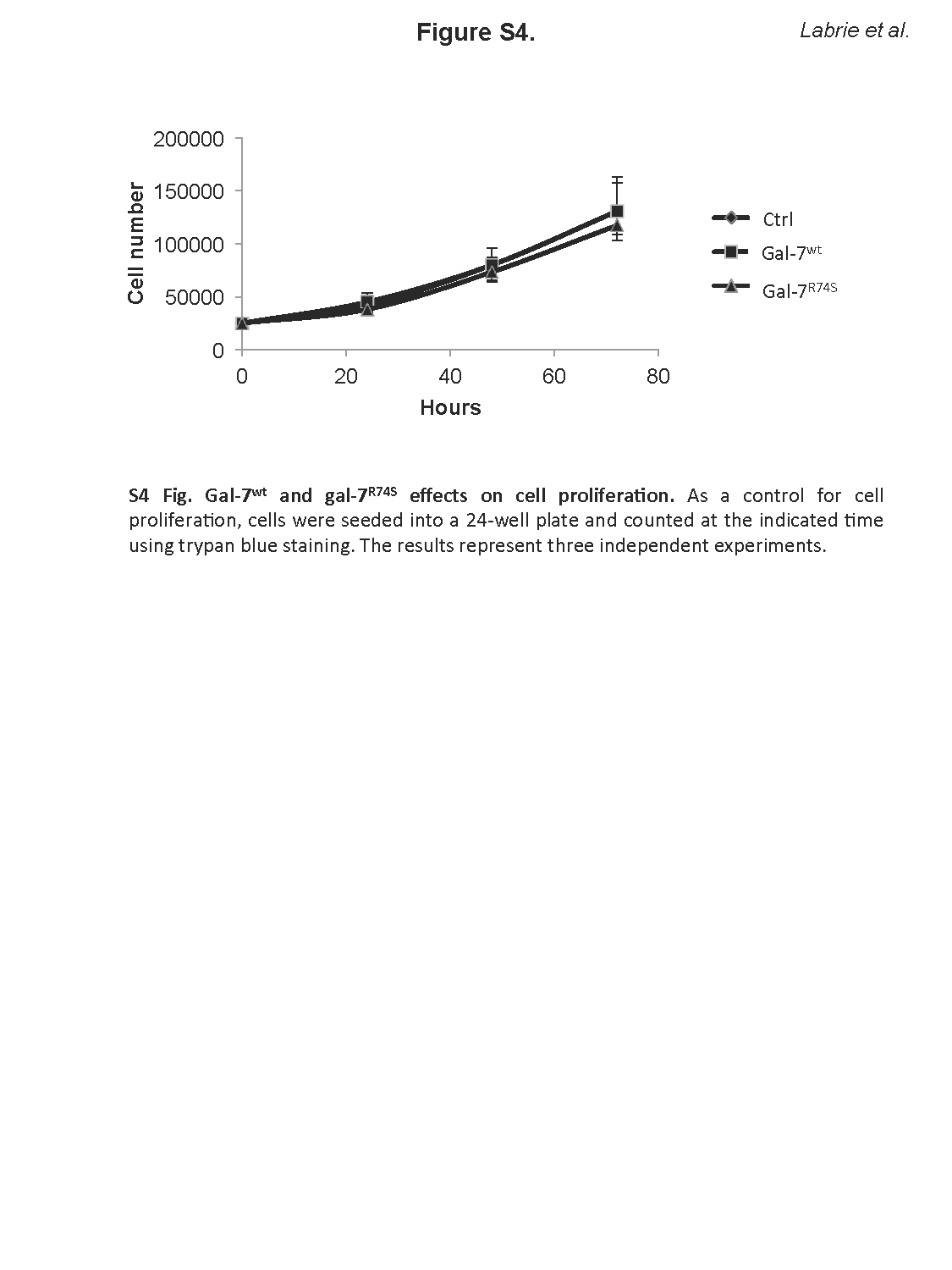

Supplement: S4 Fig — As a control for cell proliferation, cells were seeded into a 24-well plate and counted at the indicated time using trypan blue staining. The results represent three independent experiments. (TIFF) [file pone.0131307.s004.tiff]

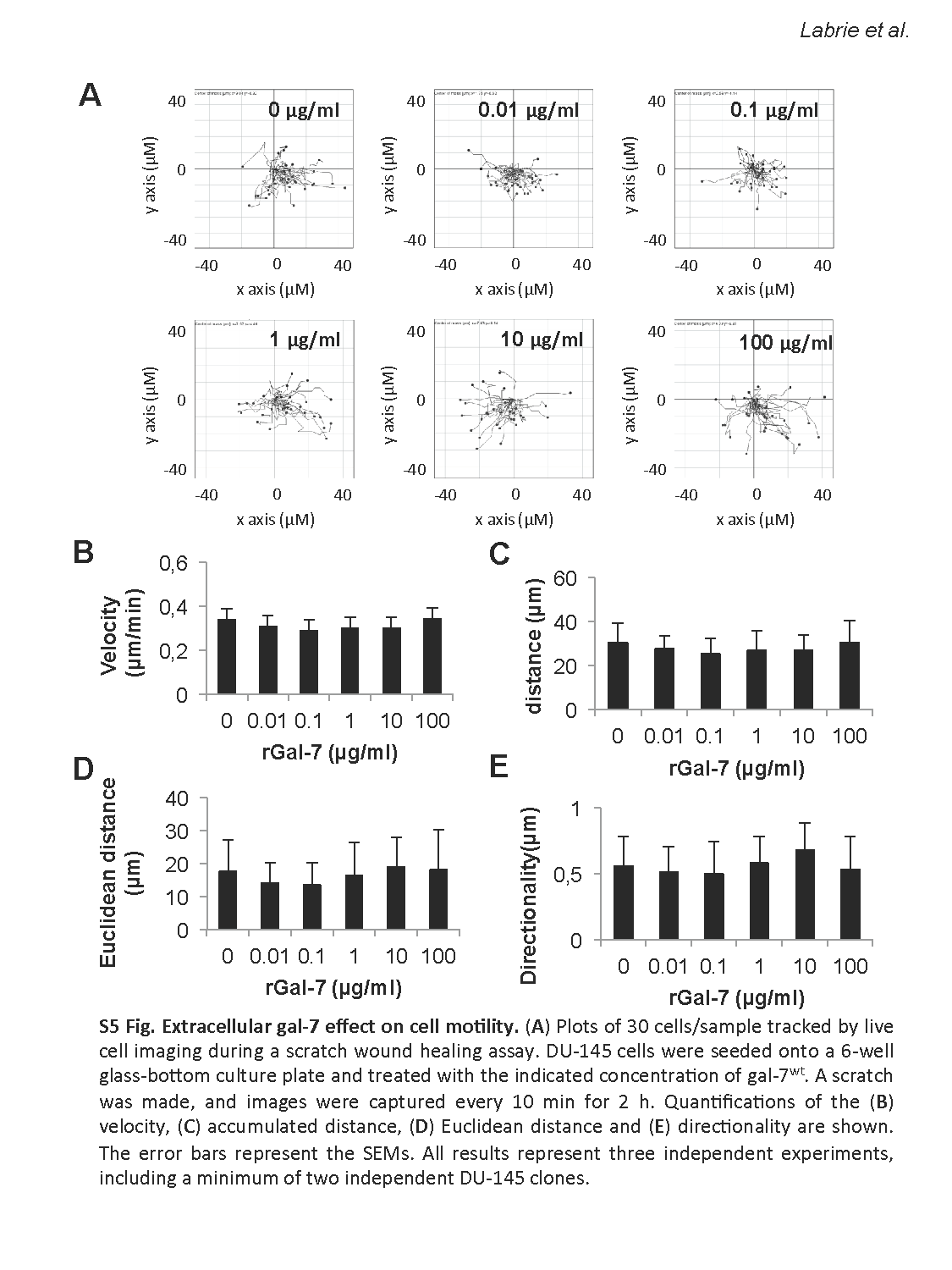

Supplement: S5 Fig — (A) Plots of 30 cells/sample tracked by live cell imaging during a scratch wound healing assay. DU-145 cells were seeded onto a 6-well glass-bottom culture plate and treated with the indicated concentration of gal-7wt. A scratch was made, and images were captured every 10 min for 2 h. Quantifications of the (B) velocity, (C) accumulated distance, (D) Euclidean distance and (E) directionality are shown. The error bars represent the SEMs. All results represent three independent experiments, including a minimum of two independent DU-145 clones. (TIFF) [file pone.0131307.s005.tiff]

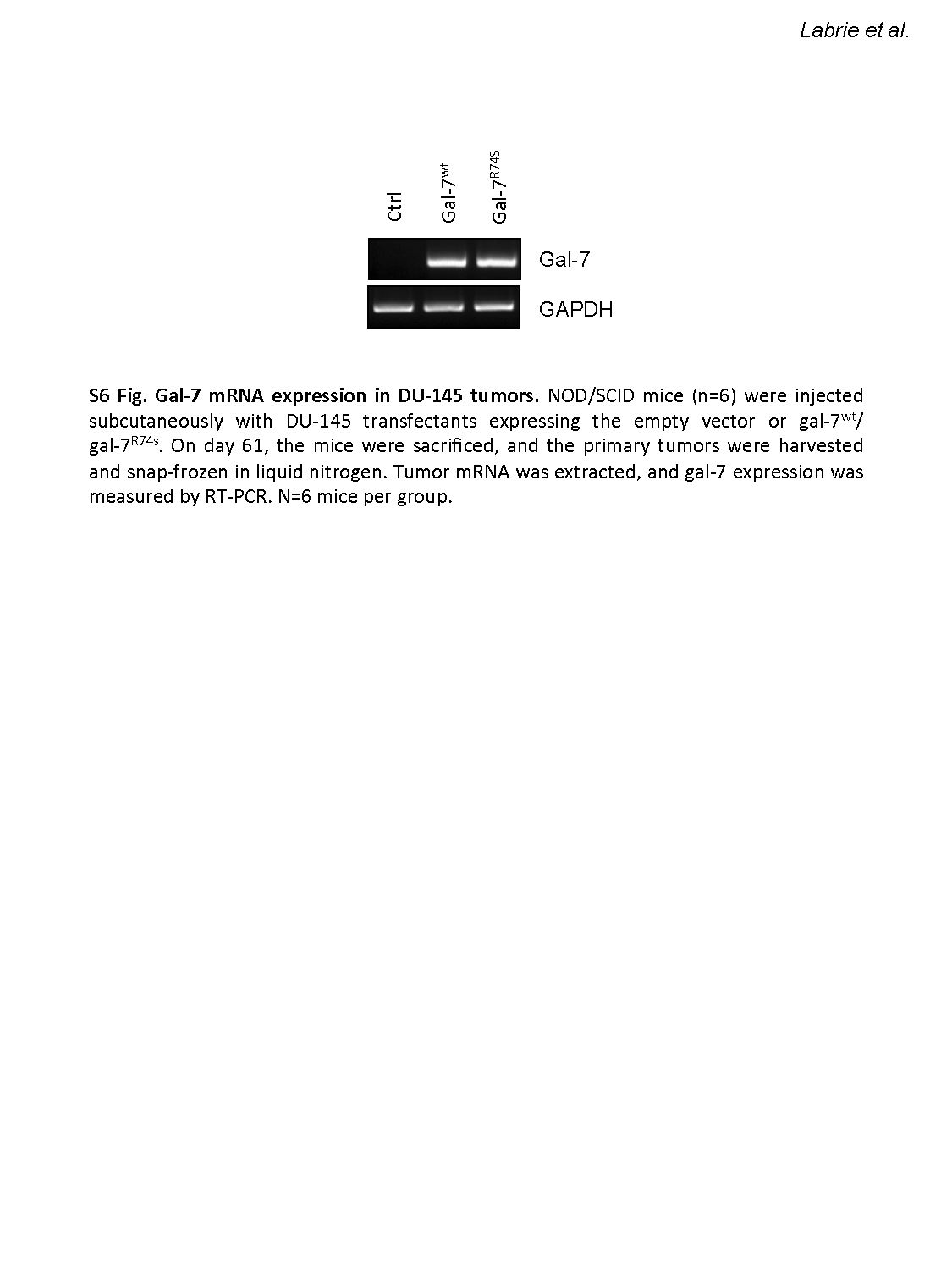

Supplement: S6 Fig — NOD/SCID mice (n = 6) were injected subcutaneously with DU-145 transfectants expressing the empty vector or gal-7wt/gal-7R74s. On day 61, the mice were sacrificed, and the primary tumors were harvested and snap-frozen in liquid nitrogen. Tumor mRNA was extracted and gal-7 expression was measured by RT-PCR. N = 6 mice per group. (TIFF) [file pone.0131307.s006.tiff]
